# Molecular and Cellular Basis of Oral Lichen Planus: Bridging Pathogenesis and Modern Clinical Paradigms

**DOI:** 10.3390/ijms27083444

**Published:** 2026-04-12

**Authors:** Kenichi Kumagai, Yuta Kishi, Taiki Suzuki, Akihisa Horie, Koji Kawaguchi, Yoshiki Hamada

**Affiliations:** 1 Department of Oral-Maxillofacial Surgery and Orthodontics, The University of Tokyo Hospital, 7-3-1 Hongo, Bunkyo-ku, Tokyo 113-8655, Japan; 2 Department of Oral and Maxillofacial Surgery, School of Dental Medicine, Tsurumi University, 2-1-3 Tsurumi, Tsurumi-ku, Yokohama 230-8501, Japan; kawaguchi-k@tsurumi-u.ac.jp (K.K.); hamada-y@tsurumi-u.ac.jp (Y.H.); 3 Department of Oral and Maxillofacial Surgery, Kanto Rosai Hospital, 1-1 Kizukisumiyoshicho, Kawasaki 211-8510, Japan; suzukitaiki000@gmail.com (T.S.); horiea@kantoh.johas.go.jp (A.H.); 4 Department of Oral and Maxillofacial Surgery, Shonan East General Hospital, 500 Nishikubo, Chigasaki 253-0083, Japan; kishi.oms@gmail.com

**Keywords:** oral lichen planus, T cell immunity, EGFR signaling, TCR repertoire, Candida, tacrolimus, Hange-shashin-to, oral potentially malignant disorder, precision oral medicine

## Abstract

Oral lichen planus (OLP) is a chronic, T cell-mediated inflammatory disorder classified by the World Health Organization as an oral potentially malignant disorder (OPMD). Despite decades of research, its precise etiology remains incompletely understood and involves a complex interplay between genetic predisposition, environmental triggers, and autoimmune-like responses. This review provides a comprehensive update on OLP pathogenesis, emphasizing the role of CD8 positive cytotoxic T lymphocyte-driven basal keratinocyte apoptosis and the skewing of the T-cell receptor (TCR) repertoire. We highlight the significance of the epidermal growth factor receptor (EGFR) signaling pathway as a molecular bridge between chronic inflammation and epithelial proliferation. Furthermore, we discuss a stepwise therapeutic approach that prioritizes the management of the oral microenvironment—specifically Candida colonization and periodontal health—before escalating to immunosuppressive agents. Finally, we explore emerging precision medicine frontiers, including IL-17/IL-23 inhibitors and JAK inhibitors, alongside traditional Japanese Kampo medicine (Hange-shashin-to) and systemic adjuncts like Cepharanthine, offering a contemporary perspective on modern OLP management. This integrative framework redefines OLP not merely as a chronic inflammatory disorder, but as an immunologically sustained, microenvironment-driven, potentially malignant condition.

## 1. Introduction

Oral lichen planus (OLP) is a chronic inflammatory mucocutaneous disease affecting approximately 0.5–2.0% of the global population, with a noted predilection for middle-aged females [1,2]. Unlike its cutaneous counterpart, which is often self-limiting, OLP is characterized by a recalcitrant clinical course and is categorized as an oral potentially malignant disorder (OPMD), with a malignant transformation rate to oral squamous cell carcinoma (OSCC) estimated between 1% and 3%. Despite decades of research, its precise etiology remains incompletely understood and involves a complex interplay between genetic predisposition, environmental triggers, and autoimmune-like responses [3].

The landscape of OPMDs has been significantly redefined in the WHO Classification of Head and Neck Tumours (5th Edition) [4]. This revision integrates “precancerous lesions” and “precancerous conditions” into a unified OPMD framework, explicitly adding oral lichenoid lesions (OLL) and oral graft-versus-host disease (OGvHD) as distinct categories. One of the most persistent challenges in oral medicine is the clinicopathological differentiation between idiopathic OLP and OLL. The WHO 5th Edition now formally recognizes OLL as a distinct OPMD category, acknowledging its unique clinical triggers and potentially higher malignant transformation rate compared to true OLP. Identifying these molecular and clinical signatures is crucial, as the therapeutic resolution of OLL hinges on the removal of external triggers, whereas OLP requires long-term immune modulation. In contrast, the term oral lichenoid reaction (OLR) is often used to describe lesions with identifiable etiological triggers, such as medications or dental materials, emphasizing their reactive and immunologically induced nature. In this review, we use OLL as a diagnostic category and OLR to denote trigger-associated immune-mediated conditions.

Following the WHO Classification of Head and Neck Tumours (5th edition, 2022) and recent domestic consensus, clinicians must now recognize that severe oral epithelial dysplasia (OED) in OLP-like lesions should be managed synonymously with carcinoma in situ (CIS). Prompt intervention is warranted in these high-risk cases to prevent progression to invasive OSCC.

The pathogenesis of OLP is primarily driven by an antigen-specific T cell-mediated immune response directed against basal keratinocytes [5,6]. The clinical management of OLP remains a formidable challenge, often characterized by a protracted course and high symptom burden. While conventional therapies focus on symptom suppression, a growing gap exists between emerging molecular insights and everyday clinical practice. To provide clinicians with a biologically rational strategy to optimize treatment outcomes and minimize iatrogenic risks in refractory cases, this review aims to bridge this gap by integrating recent advancements in T-cell biology and microenvironmental factors into a structured therapeutic framework.

## 2. Pathogenesis: The Molecular and Cellular Basis

### 2.1. The Molecular Frontier of Differential Diagnosis: OLP vs. OLL

It is clinically imperative to distinguish idiopathic OLP from OLL. While both share similar histopathological features, OLL is characterized by a more prominent B-cell infiltration and the frequent formation of tertiary lymphoid structures (TLS) compared to the T-cell-dominant environment of OLP. From a therapeutic perspective, OLL requires the identification and removal of specific triggers (e.g., dental materials or systemic drugs), whereas OLP necessitates long-term, multi-faceted immune modulation. This implies a stronger humoral immune component in OLL, necessitating therapeutic strategies focused on trigger removal rather than long-term immunomodulation [7].

### 2.2. T-Cell-Mediated Cytotoxicity and TCR Repertoire in OLP

The hallmark of OLP is a dense, band-like infiltrate of T lymphocytes in the superficial lamina propria. CD8^+^ cytotoxic T lymphocytes (CTLs) are the primary effectors of basement membrane damage, inducing basal keratinocyte apoptosis through the secretion of granzyme B/perforin and the activation of the Fas/FasL pathway [8].

**Figure 1:**
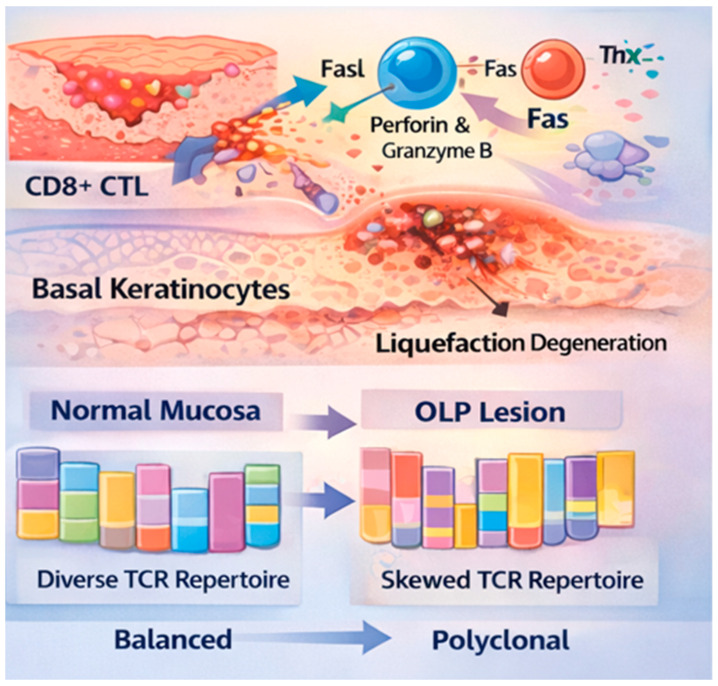
Immunological Mechanism of T Cell-Mediated Cytotoxicity and TCR Repertoire Skewing in OLP.

The investigation of T-cell receptor (TCR) clonality has evolved significantly over the past decades. Early studies by Simark-Mattsson et al. first identified a biased usage of VA2 and VB3 families in OLP lesions [9], while subsequent research by Wadehra et al. emphasized the dominance of VB22 and VB23 [10]. Our studies have demonstrated a skewing of TCR usage, specifically the overrepresentation of certain V alpha and V beta gene families (e.g., TCR VA8-1, VA22-1, and VB2-1) within the lesions [11]. Crucially, these expansions are polyclonal, suggesting that the immune response is not a monoclonal malignancy but a coordinated reaction to multiple autoantigens or microbial superantigens (Figure 1). Recent studies have contributed to this field by elucidating the crosstalk between these infiltrating T cells and the oral epithelium, particularly focusing on the role of regulatory T cells (Tregs) and their dysregulation in sustaining the chronic inflammatory state [12]. Furthermore, modern single-cell immune profiling has confirmed that these expanded T-cell clones are often in a state of functional exhaustion, yet they maintain a complex polyclonal architecture, reinforcing the hypothesis of a multi-antigenic or superantigenic drive in OLP pathogenesis [13].

Modern single-cell analyses have also revealed that many expanded CD8^+^ T cells in OLP exhibit a terminally exhausted phenotype (Tex), co-expressing PD-1, LAG-3, and TIGIT while showing reduced cytotoxic transcripts [14]. In parallel, clonally expanded tissue-resident memory T cells (TRM; CD69^+^CXCR6^+^) accumulate adjacent to the epithelium—particularly in erosive OLP—where they secrete IFN-γ, TNF-α, and IL-17, amplifying local inflammation [15]. Unlike earlier bulk TCR analyses that demonstrated skewed polyclonality, recent single-cell approaches have resolved functional heterogeneity within expanded clones, revealing exhaustion and tissue residency as key drivers of persistence. Regulatory T cells are present but functionally impaired; intriguingly, an increased population of IL-35–producing induced Tregs (iTr35) has been detected in OLP patients, suggesting a compensatory yet insufficient attempt to restrain inflammation. These findings suggest that chronic antigenic stimulation leads to T-cell dysfunction, which may explain the persistent, non-resolving nature of the inflammation.

The pathogenesis of OLP is driven by an antigen-specific immune response. CD8^+^ CTLs target basal keratinocytes, inducing apoptosis through the release of granzyme B and perforin, as well as Fas/FasL interactions. Th1 and Th17 cytokines (IFN-gamma, TNF-alpha, and IL-17) orchestrate the inflammatory milieu and basement membrane disruption. While healthy mucosa exhibits a diverse, balanced TCR profile, OLP lesions show a skewed usage of specific TCR V alpha and V beta gene families. The T cell expansion in OLP remains polyclonal (indicated by multiple-colored segments within each bar), implying response to complex antigens rather than monoclonal proliferation.

### 2.3. The EGFR Signaling Loop: From Inflammation to Proliferation in OLP

**Figure 2:**
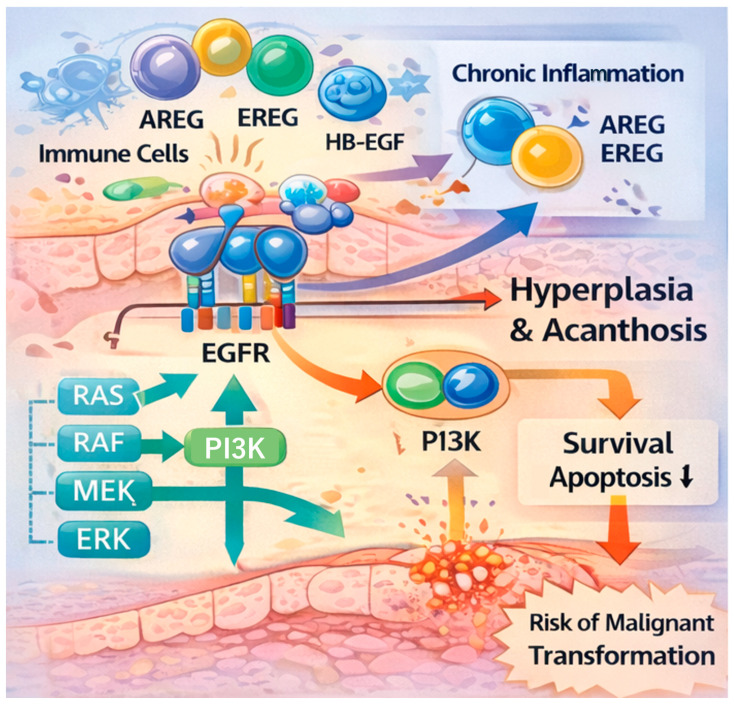
The EGFR/Ligand Autocrine and Paracrine Loop in OLP Pathogenesis.

The classification of OLP as an OPMD necessitates a deeper understanding of the molecular triggers for transformation. A key unresolved question is whether the upregulation of the EGFR and its ligands is merely a reactive response to chronic mucosal injury or a driver of oncogenesis. Our findings demonstrate that OLP lesions do not merely show transient EGFR expression. The infiltrating lymphocytes and activated keratinocytes produce EGFR ligands, including AREG, EREG, and HB-EGF, which bind to upregulated EGFR on basal/suprabasal keratinocytes. Infiltrating mononuclear cells and keratinocytes themselves produce these ligands, creating an autocrine and paracrine loop (Figure 2). We propose that this molecular signature, particularly when accompanied by secondary genetic alterations such as p53 mutations, should be incorporated into a multi-biomarker panel for risk stratification [16]. While this mechanism initially serves to maintain mucosal integrity against T cell-mediated apoptosis, the sustained mitogenic signaling may inadvertently create a pro-tumorigenic microenvironment. In this landscape, keratinocytes are more susceptible to secondary genetic alterations, such as p53 mutations or p16 inactivation. Therefore, we propose that the EGFR/ligand expression profile should be viewed as a vital component of a multi-biomarker panel for risk stratification, identifying OLP cases with a higher propensity for malignant progression.

Chronic inflammation in OLP triggers an EGFR autocrine/paracrine loop. Infiltrating lymphocytes and activated keratinocytes produce EGFR ligands, including AREG, EREG, and HB-EGF, which bind to upregulated EGFR on basal/suprabasal keratinocytes. This activation triggers downstream signaling cascades (RAS/RAF/MEK/ERK and PI3K/Akt), which drive epithelial hyperplasia, acanthosis, and potentially facilitate the progression toward malignant transformation in long-standing lesions.

### 2.4. The Paradox of Malignant Transformation: Beyond Morphological Dysplasia

A definitive diagnosis of OLP requires the total absence of epithelial dysplasia. However, long-standing OLP exhibits a paradox where morphologically benign lesions undergo malignant transformation. This process is driven by proliferative stress. The chronic T cell-mediated attack-and-repair cycle, which is facilitated by persistent EGFR signaling, induces genomic instability and secondary genetic alterations, such as p53 mutations. Consequently, OLP is classified as an OPMD not due to its initial morphology, but because its chronic inflammatory microenvironment is inherently pro-tumorigenic. This concept reframes OLP as an inflammatory field disease rather than a purely morphologic precursor lesion.

## 3. The Role of the Microenvironment

### 3.1. Secondary Candida Infection: A Critical Modifier

The presence of Candida species in OLP lesions is not merely a coincidence. The compromised mucosal barrier and the use of topical steroids create an ideal niche for fungal overgrowth [17].

- Exacerbation: Candida antigens can act as superantigens, further stimulating the T-cell infiltrate and worsening the inflammatory state.
- Diagnostic Pitfall: Secondary candidiasis can mask the underlying OLP or mimic epithelial dysplasia.

### 3.2. Periodontal Health

Poor oral hygiene and mechanical irritation from dental calculus exacerbate the “Koebner phenomenon” in the oral cavity. Chronic marginal gingivitis often synergizes with OLP (desquamative gingivitis), making resolution impossible without professional mechanical debridement.

### 3.3. Microbiome–Metabolome Interactions and Neuro-Immune Modifiers

Growing multi-omics evidence implicates oral dysbiosis as an immunologic co-driver of OLP. A recent microbiome–metabolome integration study demonstrated distinct oral microbiota profiles and metabolomic signatures in OLP, supporting a bidirectional interaction between microbial ecology and host inflammatory metabolism [18]. In addition, gut microbiome dysbiosis may contribute to systemic Treg dysregulation, which in turn sustains the chronic inflammatory state in the oral mucosa.

A landmark study further characterized salivary microbiota and metabolite alterations in OLP patients with psychiatric symptoms, suggesting that stress-associated neuro-immune changes may intersect with oral dysbiosis and mucosal immunity [19]. These datasets collectively motivate future work to test causal pathways (e.g., microbial antigen presentation, metabolite-driven Th17 skewing) and evaluate microbiome-targeted adjuncts (antifungals, periodontal therapy, probiotics) within controlled designs. Furthermore, stress-associated neuro-immune changes (e.g., Substance P release) intersect with oral dysbiosis to trigger flare-ups. Their findings suggest that stress-associated neuro-immune changes may directly interact with oral dysbiosis and mucosal immunity, highlighting the importance of psychological assessment in comprehensive OLP management.

## 4. Therapeutic Strategies: A Stepwise Approach

OLP typically manifests as bilateral, symmetrical lesions on the buccal mucosa, tongue, or gingiva. While the reticular type (Wickham’s striae) is often asymptomatic, the erosive and atrophic variants cause significant pain and a reduced quality of life [1,2].

### 4.1. Phase-Based Management: Acute vs. Chronic

Current Japanese consensus emphasizes a phase-based assessment to determine therapeutic intensity [20]:

- Acute Phase: Characterized by rapid onset and intense pain.
- Chronic Active Phase: Persistent erosions requiring continuous modulation.
- Chronic Non-active Phase: Presence of reticular striae without symptoms; the primary goal for long-term malignancy surveillance.

### 4.2. Stepwise Management

Management must be individualized based on the clinical subtype and symptom severity. Our proposed five-step protocol aligns with established guidelines, such as the American Academy of Oral & Maxillofacial Pathology (AAOMP) position paper and the Japanese OLP consensus. However, it is distinguished by its foundational emphasis on microenvironmental stabilization. By prioritizing the control of Candida species and periodontal inflammation before escalating to potent immunosuppressants, this induction-first approach effectively clears secondary inflammatory noise—a process we term the unmasking phenomenon, thereby allowing for more precise clinical grading. Hence we propose the following five-step protocol (Table 1):

### 4.3. Calcineurin Inhibitors: Tacrolimus

Tacrolimus (FK506) acts by binding to FKBP12, inhibiting calcineurin, and subsequently preventing the dephosphorylation of NFAT (nuclear factor of activated T-cells). This leads to a profound suppression of IL-2 and other Th1 cytokines. It is particularly effective for erosive OLP that has failed to respond to clobetasol [21]. Tacrolimus (0.1%) is not currently covered by national health insurance for the treatment of OLP in Japan. However, its therapeutic efficacy and safety have been reported in international clinical trials [22]. Available evidence suggests that tacrolimus is an effective second-line option for refractory erosive OLP. However, clinicians in Japan must consider its off-label status and monitor for transient local irritation or potential systemic absorption during long-term use.

### 4.4. Japanese Kampo Medicine: Hange-Shashin-to (TJ-14)

Hange-shashin-to (TJ-14) is widely used in Japan for chemotherapy-induced stomatitis and is frequently utilized as an adjunctive therapy for OLP [23].

In the therapeutic strategy for OLP in Japan, Hange-shashin-to (TJ-14) is clinically positioned as a significant adjunctive or alternative therapy, particularly for patients with symptomatic erosive or ulcerative lesions. TJ-14 is practically covered by national health insurance under the broader diagnosis of stomatitis. This formal indication effectively covers various inflammatory mucosal conditions, including both symptomatic OLP and chemotherapy-induced oral mucositis [23]. Crucially, the clinical application of Kampo medicine relies not only on Western medical diagnosis but also on the assessment of “Sho” (the patient’s unique constitutional and symptomatic pattern). For TJ-14, the diagnosis of “Sho” typically involves epigastric distress, nausea, and a tendency toward diarrhea. Aligning the treatment with the patient’s specific “Sho” is essential to maximize therapeutic efficacy and minimize adverse effects [6,23].

TJ-14 and its active ingredients, such as baicalin from Scutellariae Radix, significantly inhibit cyclooxygenase-2 (COX-2) expression and reduce the production of prostaglandin E_2 (PGE_2) in oral keratinocytes [24,25]. This suppression of the inflammatory cascade is further supported by its ability to inhibit the chemotaxis of inflammatory cells, thereby mitigating mucosal tissue damage [26,27].

Beyond anti-inflammatory and analgesic effects, TJ-14 promotes mucosal integrity and defense [28]. Furthermore, TJ-14 has been shown to facilitate tissue repair by promoting the migration of oral keratinocytes through the activation of the mitogen-activated protein kinase (MAPK) pathway and CXCR4 signaling [29]. Additionally, its free radical scavenging properties provide a cytoprotective effect against oxidative stress-induced mucosal injury [30]. Collectively, these findings demonstrate that TJ-14 is considered to act as a multi-target immunomodulator that stabilizes the oral microenvironment, supporting its potential role as an adjunctive therapy for OLP. While Kampo medicine, such as TJ-14, has shown clinical efficacy in Japan [23], its global generalizability is limited by the lack of standardized international trials and variability in evidence quality across different populations. Further multi-center validation is required to establish its role in global OLP paradigms.

### 4.5. Systemic Adjunctive Therapy: Cepharanthine

In Japanese clinical practice, Cepharanthine, a bisbenzylisoquinoline alkaloid, is frequently utilized as a systemic adjunctive therapy for OLP. It is administered with the expectation of enhancing local microcirculation through its vasodilatory properties and providing immunomodulatory effects. Retrospective multicenter data from Japan suggest potential clinical benefit, although high-level evidence remains limited [31].

## 5. Emerging Perspectives: Precision Medicine in OLP

### 5.1. New Frontiers in OLP Therapy: Molecularly Targeted Agents and Stromal Modulation

The future of OLP therapy lies in molecularly targeted agents. Recent studies have highlighted the IL-23/IL-17 axis as a major driver of the chronic erosive state. Furthermore, the discovery of inflammatory fibroblast subsets through single-cell analysis—which produce CXCL10 and CXCL12 to recruit CXCR3+ T cells—opens new avenues for therapeutic targeting of the stromal-immune crosstalk, rather than focusing solely on the lymphoid compartment. These data support the concept that OLP behaves as a Th17-driven mucosal inflammatory disease in a subset of patients.

- IL-17 Inhibitors: IL-17 pathway blockade has emerged as a biologically plausible therapeutic strategy for lichen planus. Secukinumab has been evaluated in a randomized controlled trial for lichen planus; however, the primary endpoint was not met, and the efficacy appeared to vary across disease subtypes [32].
- JAK Inhibitors: By blocking the JAK-STAT pathway, these agents can simultaneously inhibit multiple cytokine signals (IFN-gamma, IL-6, IL-2). These agents may function as a broad immunomodulatory strategy [3].

Despite the promising results of IL-17 and JAK inhibitors in recent reports, several limitations must be acknowledged. Most available data are derived from small-scale or proof-of-concept studies, and large-scale randomized controlled trials (RCTs) are currently lacking. Furthermore, the high economic burden and potential risks of systemic infection necessitate a cautious, case-by-case approach in clinical application.

### 5.2. Immune Checkpoints and Oral Lichenoid Reactions

Immune checkpoint pathways provide a clinically informative lens for OLP immunobiology. Oral lichenoid reactions are recognized as immune-related adverse events of PD-1/PD-L1 blockade; these lesions phenocopy OLP/OLL histology and offer an in vivo demonstration that releasing checkpoint brakes can precipitate interface mucositis. A mechanistic review of lichen planus associated with immune checkpoint inhibitors summarizes clinical patterns and implicates dysregulated PD-1 axis signaling in lichenoid eruption development [33]. Such observations reinforce the hypothesis that checkpoint insufficiency or imbalance may contribute to persistent cytotoxic inflammation in idiopathic OLP, while also cautioning clinicians managing oral mucosal immune-related adverse events (irAEs) to coordinate with oncology teams when systemic immunosuppression is considered.

### 5.3. Targeted Systemic Therapies: Evidence and Practical Positioning

Targeted therapies for severe, refractory disease are transitioning from case series to higher-quality evidence. A randomized placebo-controlled trial evaluated an IL-17 pathway strategy in lichen planus, reinforcing the biologic plausibility of Th17 blockade in erosive disease and providing a template for endpoint selection (pain, erosive area, relapse, safety) [32]. Observations of lichenoid eruptions following PD-1/PD-L1 blockade highlight the checkpoint axis’s role in mucosal immune homeostasis [33]. Clinical trials of IL-17 blockade such as secukinumab have begun to assess efficacy in mucosal and cutaneous lichen planus, providing a proof-of-concept for Th17-centric therapeutic strategies [32]. In parallel, recent reviews of lichen planus pathogenesis and clinical trials synthesize the expanding landscape of IL-17/IL-23 inhibitors, JAK inhibitors, and emerging small molecules (including TYK2 inhibitors), offering practice-oriented algorithms for when systemic therapy is justified [34]. Given OLP’s chronic course and OPMD status, systemic options should be positioned within a risk–benefit framework that accounts for symptom burden, disease extent, steroid-refractory status, comorbidities (e.g., diabetes, infection risk), and the need for longitudinal cancer surveillance.

## 6. Conclusions

Oral lichen planus is a complex immunological disease where the soil (the oral microenvironment) is as important as the seed (the T-cell infiltrate). A therapeutic paradigm that begins with the management of Candida and periodontal health, followed by targeted suppression of the T-cell response and growth factor signaling, offers the best chance for symptomatic control. Given its status as an OPMD, long-term surveillance remains the cornerstone of care. Future research with molecular and cellular modulation in OLP may provide the next generation of treatments for this challenging condition.

## Figures and Tables

**Table 1:** Stepwise Management of Oral Lichen Planus.

Step	Phase	Intervention	Rationale
1	Foundational	Professional oral cleaning, anti-fungal therapy (e.g., Miconazole).	Eliminates oral microbial triggers and reduces the effect of periodontitis.
2	First-line	Topical Corticosteroids (e.g., Clobetasol, Triamcinolone).	Broad anti-inflammatory effect; suppresses T-cell activity.
3	Second-line	Topical Calcineurin Inhibitors (e.g., Tacrolimus 0.1%).	Targets IL-2 production; effective in steroid-refractory cases.
4	Adjunctive	Kampo Medicine (Hange-shashin-to, TJ-14).	Reduces PGE2; provides antioxidant and anti-inflammatory support.
5	Advanced	Biologics (Anti-IL-17, JAK inhibitors).	Targeted precision medicine for recalcitrant, severe disease.

## Data Availability

No new data were created or analyzed in this study. Data sharing is not applicable to this article.

## Abbreviations

The following abbreviations are used in this manuscript:

OLP	Oral lichen planus
OPMD	Oral potentially malignant disorder
TCR	T-cell receptor
EGFR	Epidermal growth factor receptor
OSCC	Oral squamous cell carcinoma
OLL	Oral lichenoid lesions
OGvHD	Oral graft-versus-host disease
OED	Oral epithelial dysplasia
CTLs	cytotoxic T lymphocytes
Tex	Terminally exhausted phenotype
TRM	Tissue-resident memory T cells
Tregs	Regulatory T cells
